# Diagnostic and prognostic usefulness of mid-regional pro-adrenomedullin levels in patients with severe sepsis

**DOI:** 10.1186/2197-425X-3-S1-A306

**Published:** 2015-10-01

**Authors:** F Valenzuela Sanchez, B Valenzuela Mendez, R Bohollo de Austria, JF Rodríguez Gutierrez, M Jaen Franco, MA González García, A Jareño Chaumel

**Affiliations:** Hospital del SAS de Jerez, Critical Care Medicine, Jerez de la Frontera, Spain; Hospital Universitari Germans Trias i Pujol, Obstetric and Ginecology Department, Barcelona, Spain; Hospital del SAS de Jerez, Hematology Jerez de la Frontera, Spain; Hospital del SAS de Jerez, Clinical Analysis Laboratory, Jerez de la Frontera, Spain

## Introduction

Mid-regional pro-adrenomedullin (MR-proADM) is a fragment of 48 amino acids which splits from the final proADM molecule in a ratio of 1:1 with ADM. It is essentially irrelevant, but proportionally represents the levels and activity of ADM. Its half-life is several hours longer, and its plasma concentrations can be determined in clinical practice. It has been identified as a prognostic marker, stratifying the mortality risk in patients with sepsis.

## Objectives

To evaluate the usefulness of MR-proadrenomedullin (MR-proADM) levels in the diagnosis and prognosis of sepsis in patients admitted to the ICU.

## Methods

Prospective observational single-center study. A total of 120 consecutive patients with suspected severe sepsis were recruited to the ICU of Jerez Hospital. Epidemiological, clinical, laboratory data and MR-proADM, Procalcitonin (PCT), and C-reactive protein (CRP) levels were collected at the time of admission, at 48 hours, at the 5th day and on the day of discharge from the ICU.

## Results

**Figure 1:**
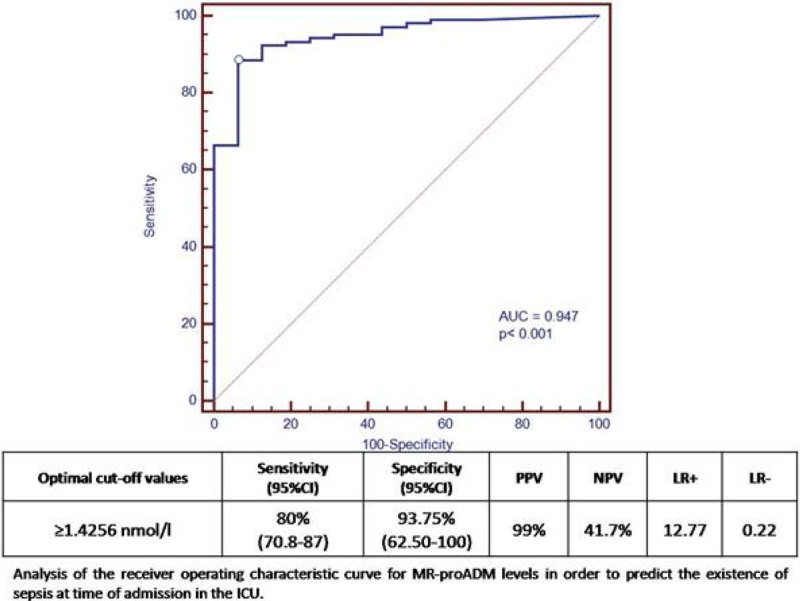
**ROC curve MR proADM sepsis diagnosis.**

104 patients were diagnosed at discharge of severe sepsis and 16 patients were diagnosed of SIRS without sepsis. The group of septic patients reached MR-proADM levels of 4.05 nmol/l vs of 0.309 nmol/l in not septic patients (p < 0.0001). The AUC-ROC was 0.9474 (Figure 1).

At 48 hours after admission the MR-proADM levels in surviving septic patients fell to 1.65 nmol/l and in the non-survivors 2.475 nmol/l (p = 0.04). On the 5th day following admission the survivor levels fell to 1.36 nmol/l vs 3.42 nmol/l in the septic patients who died in the ICU (p = 0.0006). At the 5th day the survivors showed greater clearance MR-proADM with a median level of 62.7% *vs* 21.2% in the non-survivors (Table 1).

**Table 1:** MR-ADM levels and clearance in survival subgroups

	SEPTIC SURVIVORS	SEPTIC SURVIVORS	SEPTIC NON SURVIVORS	SEPTIC NON SURVIVORS	
**MR-proADM**	median	IQR	median	IQR	***p***
ADMISSION levels (nmol/l	3.04	1.67-6.8	5.7	1.81-7.79	***p = 0.263***
48 HOURS levels (nmol/l	1.65	1.11-3.62	2.47	1.43-6.48	***p = 0.04***
5TH DAY levels (nmol/l	1.36	0.88-1.96	3.42	2.18-10.57	***p = 0.0006***
DISCHARGE levels (nmol/l)	1.268	0.88-1.78			
48 HOURS Clearance (%)	43.2	14.4-60.5	22.8	-1.3-59.72	***p = 0.20***
5TH DAY Clearance (%)	62.7	46.17-82.3	21.2	-38.7-61	***p = 0.015***

**Figure 2:**
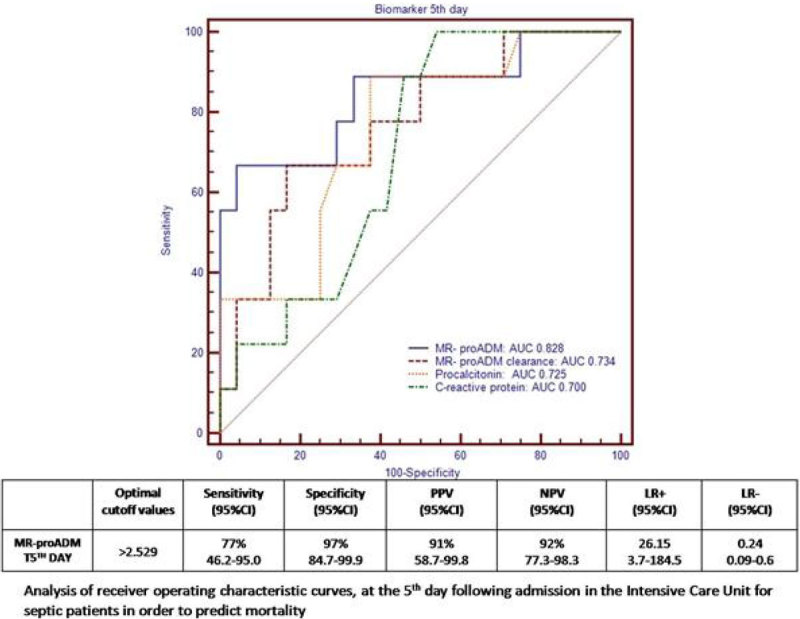
**ROC curves Biomarker 5th day prognosis.**

The AUC the ROC curve at the 5th day was: MR-proADM 0,828(p = 0,001); PCT 0.725 (p = 0,016) CRP 0.700 (p = 0,0214). The AUC the ROC curve to MR-proADM clearance at the 5th day was 0,734 (p = 0,0104) (Figure 2)

**Figure 3:**
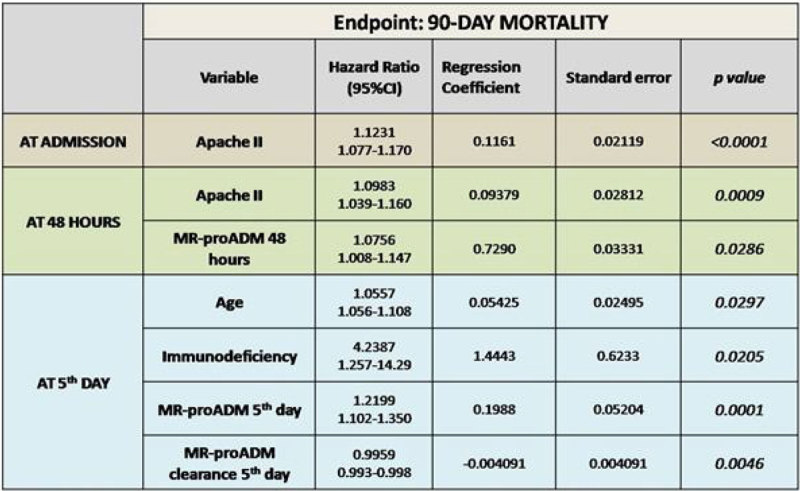
**multivariate analysis mortality 90 days.**

Patients with MR-proADM levels of 2.5 nmol/l and above or MR-proADM clearance less than 30% at the 5th day following admission in the ICU showed an enhance in mortality (p < 0.0001). In the multivariate analysis (Cox proportional hazards models) MR-proADM levels and MR-proADM clearance at the 5th day following admission, were statistically significant predictive factors for mortality in the ICU and at 90 days (Figure 3).

## Conclusions

Initial MR-proADM levels help to identify the infectious origin in patients with SIRS and organ dysfunction. MR-proADM levels and its clearance at the 5th day following admission are the most effective biomarker to determine unfavorable evolution and the risk of mortality in patients with severe sepsis admitted to the ICU.

